# Complete oxidation of hydroxymethylfurfural to furandicarboxylic acid by aryl-alcohol oxidase

**DOI:** 10.1186/s13068-019-1555-z

**Published:** 2019-09-10

**Authors:** Ana Serrano, Eva Calviño, Juan Carro, María I. Sánchez-Ruiz, F. Javier Cañada, Angel T. Martínez

**Affiliations:** 1 Biotechnology for Lignocellulosic Biomass, Centro de Investigaciones Biológicas, CSIC, Ramiro de Maeztu 9, 28040 Madrid, Spain; 2 NMR and Molecular Recognition, Centro de Investigaciones Biológicas, CSIC, Ramiro de Maeztu 9, 28040 Madrid, Spain

**Keywords:** Biocatalysis, Aryl-alcohol oxidase, Mutated variants, 5-Hydroxymethylfurfural, 5-Formylfurancarboxylic acid, Inhibition, Hydrogen peroxide, Catalase, 2, 5-Furandicarboxylic acid, Polymer building blocks

## Abstract

**Background:**

5-Hydroxymethylfurfural (HMF) is a highly valuable platform chemical that can be obtained from plant biomass carbohydrates. HMF can be oxidized to 2,5-furandicarboxylic acid (FDCA), which is used as a renewable substitute for the petroleum-based terephthalic acid in polymer production.

**Results:**

Aryl-alcohol oxidase (AAO) from the white-rot fungus *Pleurotus eryngii* is able to oxidize HMF and its derivative 2,5-diformylfuran (DFF) producing formylfurancarboxylic acid (FFCA) thanks to its activity on benzylic alcohols and hydrated aldehydes. Here, we report the ability of AAO to produce FDCA from FFCA, opening up the possibility of full oxidation of HMF by this model enzyme. During HMF reactions, an inhibitory effect of the H_2_O_2_ produced in the first two oxidation steps was found to be the cause of the lack of AAO activity on FFCA. In situ monitoring of the whole reaction by ^1^H-NMR confirmed the absence of any unstable dead-end products, undetected in the HPLC analyses, that could be responsible for the incomplete conversion. The deleterious effect of H_2_O_2_ was confirmed by successful HMF conversion into FDCA when the AAO reaction was carried out in the presence of catalase. On the other hand, no H_2_O_2_ formation was detected during the slow FFCA conversion by AAO in the absence of catalase, in contrast to typical oxidase reaction with HMF and DFF, suggesting an alternative mechanism as reported in some reactions of related flavo-oxidases. Moreover, several active-site AAO variants that yield nearly complete conversion in shorter reaction times than the wild-type enzyme have been identified.

**Conclusions:**

The use of catalase to remove H_2_O_2_ from the reaction mixture leads to 99% conversion of HMF into FDCA by AAO and several improved variants, although the mechanism of peroxide inhibition of the AAO action on the aldehyde group of FFCA is not fully understood.

## Background

Interest in the production of new chemicals and materials from renewable resources has exponentially grown in the last years with biomass-derived compounds as a future alternative to fossil-based compounds. Among them, 2,5-furandicarboxylic acid (FDCA) has been reported by the US Department of Energy as one of the top 12 value-added chemicals derived from biomass [1, 2]. Its importance mainly lies in the possibility of using FDCA as a renewable building block for the production of poly(ethylene 2,5-furandicarboxylate) (PEF) through polymerization with ethylene glycol. PEF is expected to substitute for conventional poly(ethylene-terephthalate) (PET) plastics due to its renewable origin and its better chemical and gas barrier properties [3, 4]. For these reasons, the production of FDCA from lignocellulose biomass has gained interest in the last years and different efforts were made to develop a biological methodology for FDCA synthesis, as an alternative to the chemical ones.

**Fig. 1:**
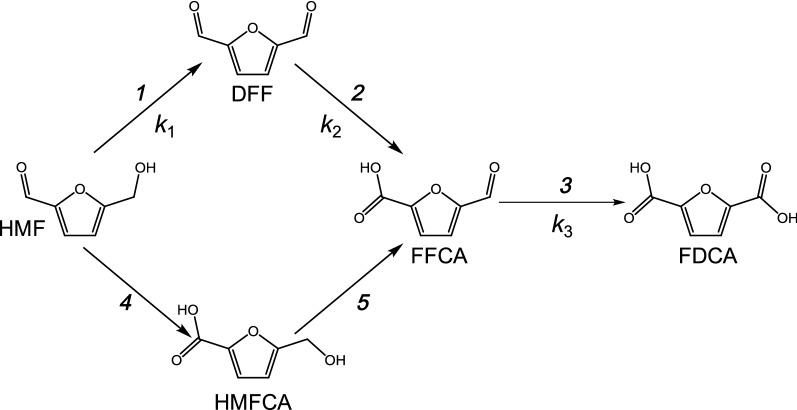
Two convergent pathways to FDCA building block from renewable HMF: The conversion starts by oxidation of the HMF alcohol (reaction 1) or aldehyde (reaction 4) groups to DFF and HMFCA, respectively, and continues with oxidation of the latter intermediates to FFCA (reactions 2 and 5, respectively) that is finally converted into FDCA (reaction 3) (the AAO rate constants, *k*_1_–*k*_3_, provided in Fig. 2 and Additional file 1: Table S1 are indicated)

FDCA production from lignocellulose biomass involves first the production of 5-hydroxymethylfurfural (HMF). This compound is obtained by dehydration of monosaccharides (generally fructose) that can originate from cellulose hydrolysis, followed by glucose isomerization [5–7]. Then, FDCA is obtained from HMF through three consecutive oxidation steps with two alternative intermediates, 2,5-diformylfuran (DFF) and 5-hydroxymethylfurancarboxylic acid (HMFCA), depending on the functional group of HMF that is oxidized first (Fig. 1, *reactions 1 and 4*, respectively). By additional oxidation, the above intermediates converge into 5-formylfurancarboxylic acid (FFCA), which is finally converted into FDCA (*reactions 2, 5, and 3*, respectively). In general, the chemical methods to obtain FDCA from HMF require high temperature, high pressure, metal catalysts, and organic solvents, which render the process polluting and expensive. For this reason, enzymatic conversion appears a suitable alternative, since enzymes are selective and act under mild conditions [8, 9].

Several enzymes are able to catalyze some of the above individual reactions in HMF conversion. On one hand, some oxidases act on the alcohol or aldehyde groups of these compounds with the only requirement of molecular oxygen as co-substrate. Among them, aryl-alcohol oxidase (AAO), a member of the glucose-methanol-choline oxidase/dehydrogenase (GMC) superfamily [10], typically oxidizes primary benzylic alcohols (i.e., those contiguous to an aromatic system) to the corresponding aldehydes [11], and hydrated aldehydes (*gem*-diols) to the corresponding acids [12] albeit the latter with lower efficiencies. In this way, the AAO from *Pleurotus eryngii* has been reported to selectively catalyze the oxidation of the hydroxyl group of HMF to produce DFF, and the subsequent oxidation of one of the aldehyde groups (hydrated as a *gem*-diol) in DFF to produce FFCA (*reactions 1 and 2*, respectively) [13]. However, its apparent inability to catalyze the full conversion into FDCA was simultaneously revealed. Moreover, galactose oxidase (GAO), a copper-radical enzyme oxidizing sugars [14], also transforms the hydroxyl group of primary benzylic alcohols into the corresponding aldehyde (as AAO does). Thus, GAO converts HMF into DFF and HMFCA into FFCA (*reactions 1 and 5*, respectively), but does not have any activity on DFF and FFCA [15]. Up to now, only one oxidase from *Methylovorus* sp., named hydroxymethylfurfural oxidase (HMFO) and also belonging to the GMC superfamily, has been reported to catalyze the total conversion of HMF into FDCA, through the DFF intermediate (*reactions 1, 2, and 3*), being the last oxidation (*reaction 3*) the limiting step for FDCA production [16, 17].

On the other hand, some heme-thiolate peroxidases/peroxygenases can hydroxylate some of these compounds at the expenses of H_2_O_2_. Chloroperoxidase (CPO) from *Leptoxyphium fumago* is able to oxidize both the alcohol and aldehyde moieties of HMF giving rise to a mixture of HMFCA, DFF, FFCA, and FDCA (by a combination of *reactions 1–5*). By dosing the amount of H_2_O_2_, the mixture can be enriched in the final product. However, this HMF oxidation is not selective and different side-products are always produced [18]. Besides, the unspecific peroxygenase (UPO) from *Agrocybe aegerita* was found to oxidize HMF into FDCA via HMFCA and FFCA (*reactions 4, 5, and 3*, respectively) with variable yields [13] by a combination of hydroxylation and dehydration reactions [19]. Recently, the alternative oxidation of HMF to FFCA via DFF (*reactions 1 and 2*) by UPO has been also reported [15]. In any case, only low amounts of FDCA are obtained upon HMF oxidation by UPO alone.

Smart combinations of the above enzymes, including AAO/CPO [18], AAO/UPO [13], GAO/UPO [20], and AAO/GAO/UPO [15], have been reported for the production of FDCA from HMF taking advantage of the fact that the by-product of oxidases, i.e., H_2_O_2_, is the co-substrate required by peroxidases/peroxygenases. However, cascades involving a combination of several enzymes have some disadvantages, such as the need to combine the different optimal operational conditions of each of them.

In the present work, we revise the lack of AAO activity on FFCA previously claimed [13], which was attributed to the low hydration degree of FFCA and/or to the deactivating effect that its carboxyl group causes on the hydride transfer reaction that characterizes AAO catalysis [21]. First, we demonstrate the ability of AAO to oxidize FFCA into FDCA. Second, we show that the control of the H_2_O_2_ levels during the reaction, by adding catalase, leads to 99% conversion of HMF into FDCA. Moreover, under such conditions, several AAO mutations at the active site are able to increase the rate of FFCA transformation into FDCA, consequently decreasing the reaction times and reaching higher conversion yields.

## Results

### FFCA oxidation as the limiting step for FDCA production

**Fig. 2:**
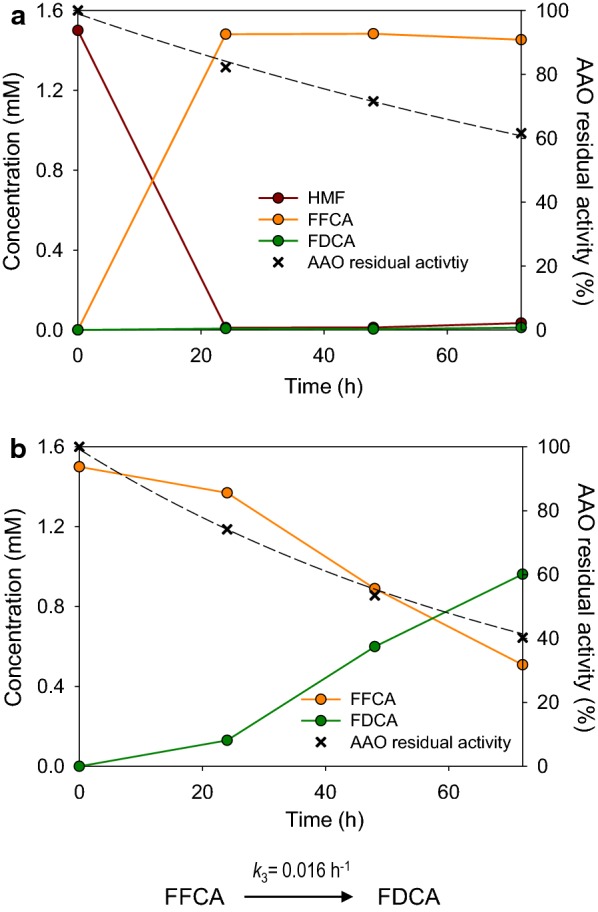
Long-term oxidations of HMF (**a**) and FFCA (**b**) by AAO showing product molar percentages referred to initial substrate concentration (continuous lines) and enzyme residual activity (dashed lines). Reactions between 1.5 mM HMF or FFCA and 2.5 µM AAO were performed in 50 mM sodium phosphate, pH 6.0, at 28 °C. Oxidation rate for FDCA production in **b**, estimated by fitting data to equation $\left[ {\text{FFCA}} \right]{ = }\left[ {\text{FFCA}} \right]_{ 0} e^{{ - k_{3} t}}$, is indicated at the bottom

**Fig. 3:**
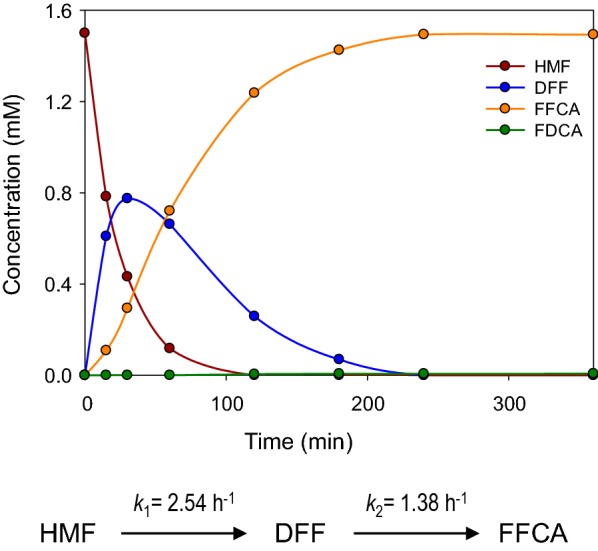
Time-course of 3-h reaction of HMF and AAO. Reactions between 1.5 mM HMF and 2.5 µM AAO were performed in 50 mM sodium phosphate, pH 6.0, at 28 °C. Oxidation rates in these conditions, estimated by fitting data to equations $\left[ {\text{HMF}} \right]{ = }\left[ {\text{HMF}} \right]_{ 0} e^{{ - k_{1} t}}$ and $\left[ {\text{DFF}} \right]{\text{ = DFF}}_{ 0} \left( {\frac{{k_{1} }}{{(k_{2} - k_{1} }}} \right)\left( {e^{ - k1t} - e^{{ - k_{2} t}} } \right)$, are indicated at the bottom

Incubation of either HMF or DFF (1.5 mM) with AAO (2.5 µM) resulted in accumulation of FFCA, without detection of FDCA after 72 h (Fig. 2a). The enzyme kept ~ 80% of its activity during the first 48 h, decreasing to 60% at the end of the 72-h reaction. Conversion of HMF into DFF was the fastest oxidation step (*k*_1_ = 2.54 h^−1^), while oxidation of DFF into FFCA was slower (*k*_2_ = 1.38 h^−1^), although accumulation of DFF was only observed during the first 30 min (Fig. 3). At this time, a mixture of HMF (29%), DFF (52%), and FFCA (19%) was found. HMF was totally consumed after 2 h, whereas its total conversion into FFCA was completed in 4 h. In contrast to previous studies [13], DFF was detected in the reaction mixture, which indicates that this compound leaves the active site before being rapidly converted into FFCA.

Surprisingly, production of FDCA was detected when FFCA was used as AAO substrate (Fig. 2b), but at a low rate (*k*_3_ = 0.016 h^−1^) compared to HMF and DFF oxidation, resulting in ~ 60% conversion after 72 h. The residual activity of AAO after this period of time represented 40% of the initial activity.

In the light of these results, it can be concluded that AAO is able to oxidize FFCA into FDCA. However, this oxidation would be clearly the limiting step for the production of FDCA from HMF, given the much lower reaction rate.

### H_2_O_2_ has a negative effect on FFCA oxidation

AAO is able to oxidize FFCA into FDCA, but this oxidation does not take place when FFCA originates from the previous oxidation of HMF and DFF. Moreover, addition of new biocatalyst after 3 h of reaction (when all HMF was converted into FFCA) did not promote oxidation of the accumulated FFCA to FDCA (data not shown). Since the only difference between these two reaction setups is the presence of two equivalents of H_2_O_2_ (formed in the subsequent oxidations of HMF and DFF), we evaluated the effect that peroxide has on both the activity and the stability of AAO.

**Fig. 4:**
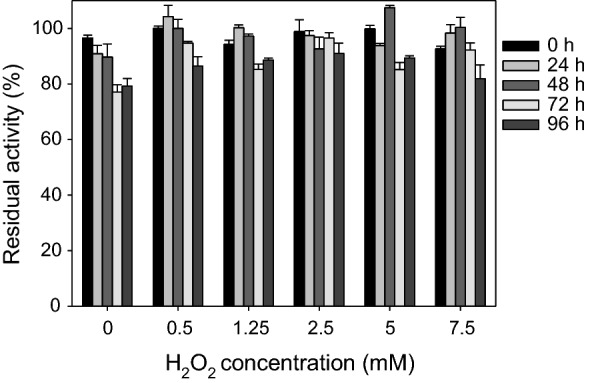
Residual activity of AAO after different times of incubation of the enzyme (~ 3 µM) with increasing amounts of H_2_O_2_ (from 0 to 7.5 mM) in 50 mM sodium phosphate, pH 6.0, at 25 °C

As shown in Fig. 4, the enzyme remained stable in the presence of increasing concentrations of H_2_O_2_ (up to 7.5 mM) maintaining at least 80% of its initial activity after 96 h of incubation at 25 °C. On the other hand, the effect of peroxide on the activity of AAO was assessed for the oxidation of its natural substrate, *p*-methoxybenzyl alcohol, and *p*-anisaldehyde [22]. The results, summarized in Table 1, indicate that the alcohol and aldehyde oxidation activities of AAO on these substrates are independent of the presence of H_2_O_2_.

**Table 1:** Kinetic parameters for the oxidation of *p*-methoxybenzyl alcohol and *p*-anisaldehyde by AAO in the presence of different H_2_O_2_ concentrations

*p*-Methoxybenzyl alcohol				*p*-Anisaldehyde	
[H_2_O_2_] (mM)	*k*_cat_ (s^−1^)	*K*_m_ (µM)	*k*_cat_/*K*_m_ (s^−1^mM^−1^)	[H_2_O_2_] (mM)	*k*_cat_ (min^−1^)
0	108 ± 3	31.8 ± 3.2	3390 ± 360	0	7.3 ± 0.7
0.5	115 ± 3	44.3 ± 3.5	2600 ± 220	1.0	7.3 ± 0.2
1.0	104 ± 3	33.5 ± 3.4	3100 ± 330	1.5	6.5 ± 0.3
1.9	109 ± 4	35.6 ± 4.3	3060 ± 390	3.1	6.7 ± 0.6
7.7	100 ± 4	33.7 ± 4.5	2970 ± 420	9.3	6.9 ± 0.5
15.5	97 ± 3	31.0 ± 2.6	3120 ± 280	12.3	6.9 ± 0.1

Activity was followed by measuring aldehyde formation (in *p*-methoxybenzyl alcohol oxidation) or acid formation (in *p*-anisaldehyde oxidation). Turnovers for aldehyde oxidation were calculated at saturating concentration of *p*-anisaldehyde (~ 5 mM) at 25 °C in air-saturated (0.256 mM O_2_) 50 mM sodium phosphate, pH 6.0

The possible effect that increasing amounts of FFCA might exert on AAO activity was also evaluated. The results indicate that FFCA does not affect the kinetic parameters for AAO oxidation of *p*-methoxybenzyl alcohol either (Table 2).

**Table 2:** Kinetic parameters for the oxidation of *p*-methoxybenzyl alcohol by AAO in the presence of different FFCA concentrations

[FFCA] (mM)	*k*_cat_ (s^−1^)	*K*_m_ (µM)	*k*_cat_/*K*_m_ (s^−1^mM^−1^)
0	84.5 ± 2.1	35.3 ± 2.8	2390 ± 200
0.4	68.0 ± 3.3	33.0 ± 5.0	2120 ± 350
0.8	78.9 ± 1.7	37.9 ± 2.5	2080 ± 140
1.5	73.8 ± 3.4	37.0 ± 5.1	1990 ± 290
3.0	66.9 ± 2.3	31.8 ± 3.6	2100 ± 250
6.0	61.9 ± 2.6	27.6 ± 2.6	2250 ± 220

Activity was followed by measuring the H_2_O_2_ released using AmplexRed^®^ and HRP at 25 °C in air-saturated (0.256 mM O_2_) 50 mM sodium phosphate, pH 6.0

**Fig. 5:**
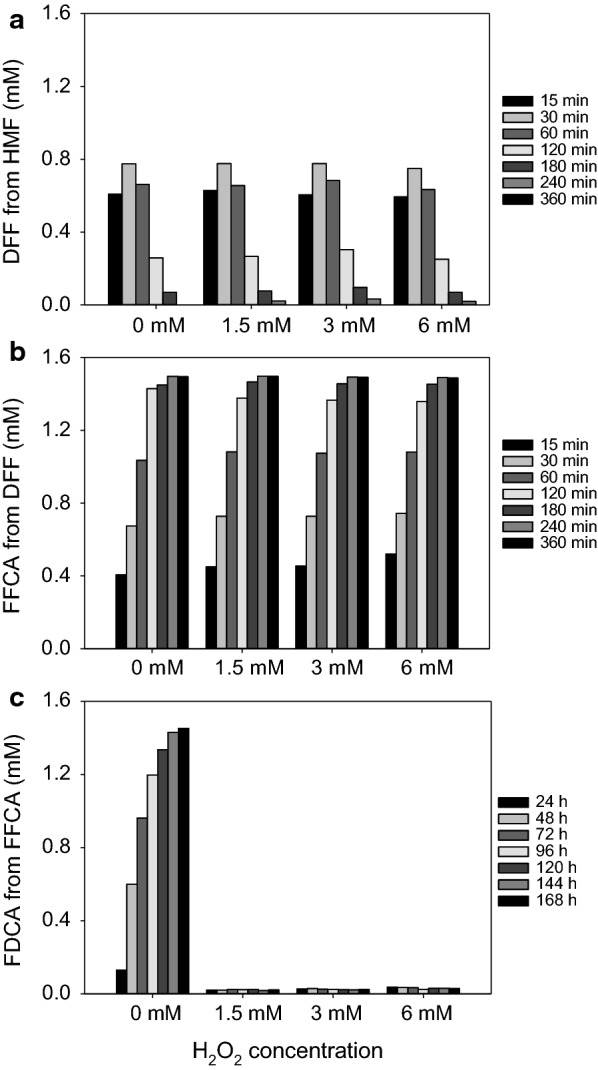
Effect of H_2_O_2_ on the oxidation of HMF (**a**), DFF (**b**), and FFCA (**c**) by AAO. Reactions between 1.5 mM HMF, DFF, or FFCA and 2.5 µM AAO were performed in 50 mM sodium phosphate, pH 6.0, at 28 °C in the presence of different amounts of H_2_O_2_

Taking into consideration that the enzyme is stable against H_2_O_2_ and its activity (in the *p*-methoxybenzyl alcohol standard assay) is not inhibited by the products of the reaction (H_2_O_2_ and FFCA), reactions on HMF, DFF, and FFCA were performed in the presence of increasing amounts of H_2_O_2_ (1.5–6 mM) to evaluate its effect. The results showed that the production of neither DFF nor FFCA was affected by peroxide (Fig. 5a, b). In all cases, the rates of product formation were similar (*k*_1_ ~ 2.5–2.7 h^−1^ and *k*_2_ ~ 1.1–1.4 h^−1^, respectively, Additional file 1: Table S1) and the enzyme kept its activity along the whole course of the reaction. However, FDCA production was abolished by H_2_O_2_ when using FFCA as substrate, even with the lowest amounts of peroxide assayed (Fig. 5c). Taking these results into account, lower H_2_O_2_ concentrations (0.0125–1.2 mM) were also evaluated for FFCA oxidation to FDCA (Additional file 1: Fig. S1). The results point out a strong inhibition of this reaction by H_2_O_2_ with an estimated half maximal inhibitory concentration (IC_50_) of ~ 158 µM. Altogether, these results indicate that only the oxidation of FFCA is somehow affected by the presence of H_2_O_2_ in the reaction mixture, at concentrations as low as 12.5 µM, and especially over 0.4 mM, under our experimental conditions.

### Complete HMF conversion by AAO in presence of catalase

To overcome the negative effect that H_2_O_2_ has on the oxidation of FFCA by AAO, catalase was added to HMF, DFF, and FFCA reactions. Since the amount of AAO was decreased to 1.5 µM in these experiments, the reactions in the presence and absence of catalase were followed for 12 days, with aliquot sampling every 24 h, trying to achieve total conversion of the substrates. The oxidized products were analyzed by HPLC, and the H_2_O_2_ formed was spectrophotometrically quantified in the reactions without catalase using the HRP-coupled assay. The AAO residual activity was also evaluated using the standard assay.

**Fig. 6:**
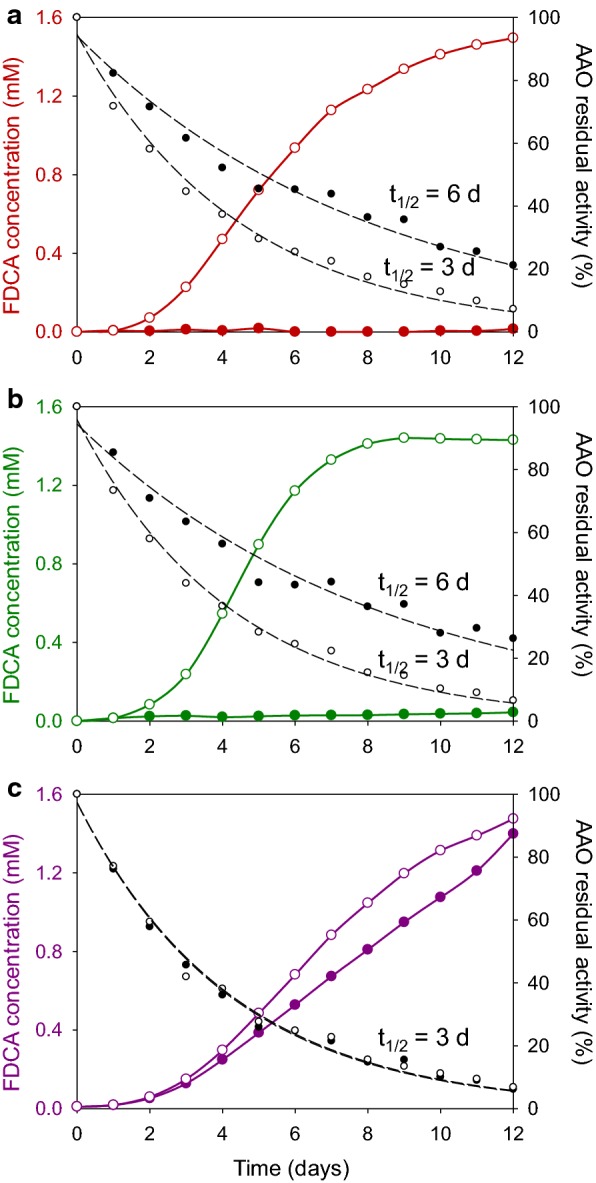
FDCA production by AAO along time in the absence (closed circles) and presence of catalase (open circles) using HMF (**a**), DFF (**b**), and FFCA (**c**) as substrate. Dashed lines represent AAO residual activity along the time course of the reaction, enabling half-life (*t*_1/2_) calculation. Reactions between 1.5 mM substrate and 1.5 µM AAO (and catalase 2–5 U/mL) were performed in 50 mM sodium phosphate, pH 6.0, at 28 °C

No FDCA production was detected from HMF or DFF in the absence of catalase, and the enzymatic activity decreased to ~ 20% at the end of the treatment (Fig. 6a, b, *closed circles*). Addition of catalase leads to the production of FDCA attaining a 100% yield after 12 days, although the final residual activity was below 10% (Fig. 6a, b, *open circles*). In the case of FFCA, the effect of catalase was negligible and a 100% FDCA yield was achieved in both conditions, keeping the enzyme ~ 10% of its initial activity (Fig. 6c).

**Fig. 7:**
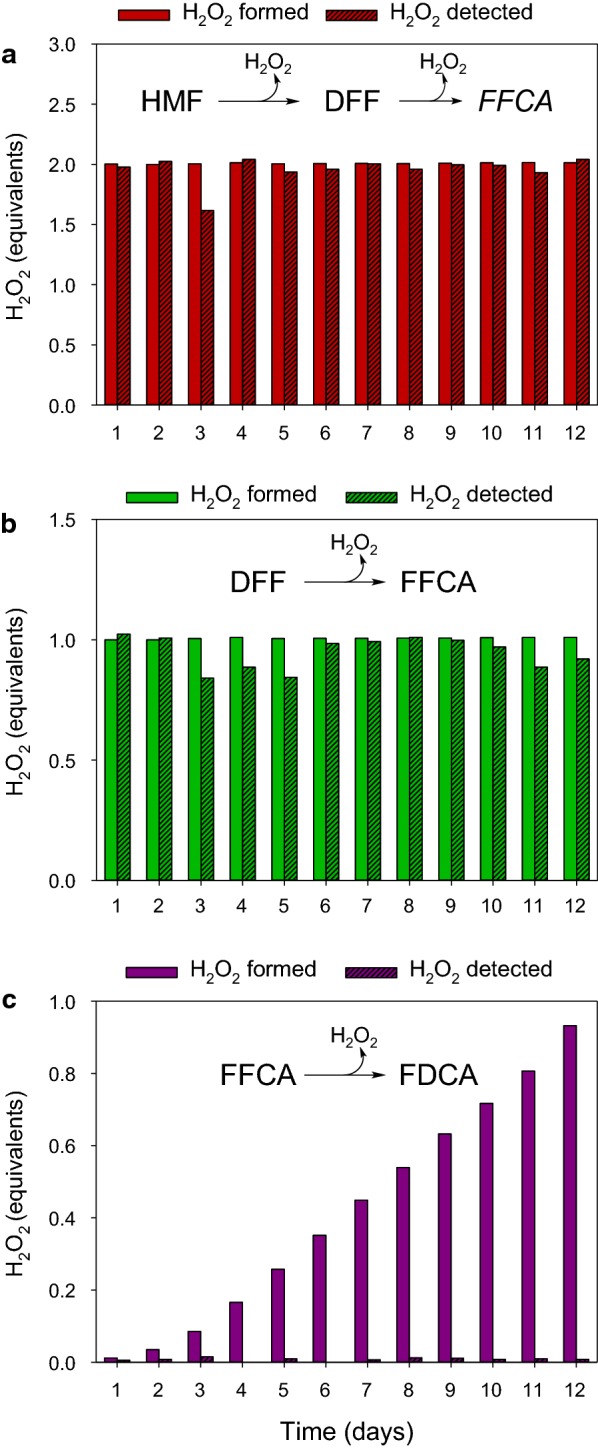
Comparison of the H_2_O_2_ equivalents detected (with AmplexRed/HRP) and the theoretical equivalents formed (according to the experimental conversion rates and the expected oxidase-type activity on all the substrates) in the reactions of HMF (**a**), DFF (**b**), and FFCA (**c**) with AAO in absence of catalase. Reactions between 1.5 mM substrate and 1.5 µM AAO were performed in 50 mM sodium phosphate, pH 6.0, at 28 °C

Based on the experimental conversions obtained in the absence of catalase, the theoretical equivalents of H_2_O_2_ formed were calculated, and compared with the amount of H_2_O_2_ detected spectrophotometrically. In the HMF and DFF reactions, the peroxide detected almost equals the theoretical one: two equivalents for the two-step oxidation of HMF into FFCA (Fig. 7a) and one equivalent for the DFF direct oxidation into FFCA (Fig. 7b). Surprisingly, no peroxide was found in the reaction mixture when FFCA was used as substrate, while, according to the progress of the reaction, one equivalent of H_2_O_2_ should be detected at the end of the experiment (Fig. 7c).

Spontaneous oxidation of FFCA and other substrates was excluded, since they remained as the most abundant compounds after 12-day incubation in the absence of AAO (~ 75–80% of the compounds detected in controls, Additional file 1: Fig. S2A–C). Since some oxidase activity (independent of H_2_O_2_) has been suggested for catalase acting on these compounds [15], controls were also incubated with this enzyme, but the results (similar to the first controls) indicate that catalase does not oxidize the substrates (Additional file 1: Fig. S2D–F).

### Analysis of HMF oxidation by AAO in the NMR tube

**Fig. 8:**
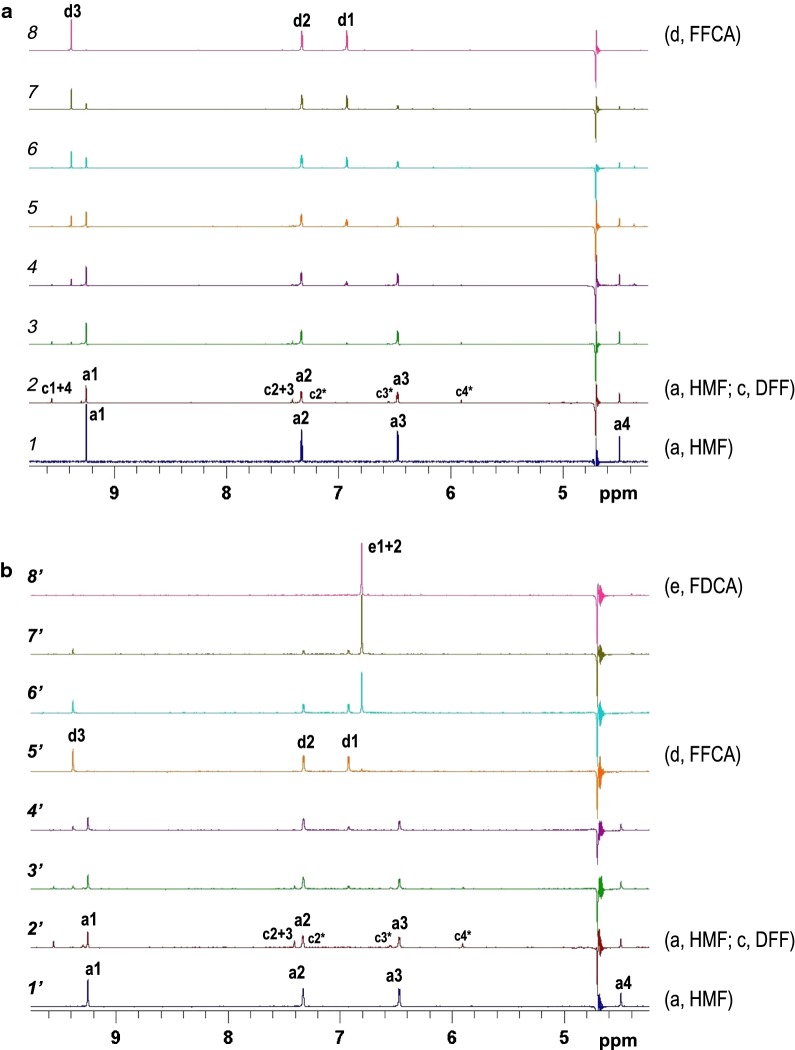
^1^H-NMR spectra along the reaction between HMF and AAO in the absence (**a**) and presence (**b**) of catalase. Spectra 1 and 1′ were obtained before enzyme addition. Spectra 2 and 2′ were recorded immediately after enzyme addition. The rest of spectra (3/3′ to 8/8′) show the evolution of the reaction products during long-term incubation at 25 °C

Taking the previous results into account, we looked into the possible formation of a dead-end FFCA product, which might be unstable under the HPLC analysis conditions (with 10 mM H_2_SO_4_ as mobile phase). Therefore, we analyzed the oxidation of HMF by AAO in real time by ^1^H-nuclear magnetic resonance (^1^H-NMR) in the NMR tube in the absence (Fig. 8a) and presence (Fig. 8b) of catalase. The aim was to identify the potential dead-end product in the HMF reactions. Under both conditions, a first spectrum of the substrate was recorded before addition of the enzyme (spectra 1 and 1′, Fig. 8). Then, both reactions were monitored according to the characteristic signals of each compound (see Additional file 1: Fig. S3 for NMR spectra of standard compounds).

Immediately after the addition of AAO (spectra 2 and 2′, Fig. 8), a decrease in the HMF signals (at 4.5, 6.4, and 9.2 ppm), together with the appearance of DFF signals (at 5.9, 7.4 and 9.5 ppm), can be observed, consistent with the fast DFF formation from HMF (Fig. 3). As the DFF signals decrease, new distinctive signals appear that correspond to the subsequent formation of FFCA (at 6.9 and 9.4 ppm) under both conditions (spectra 3 and 3′, Fig. 8). In the absence of catalase, the evolution of the spectra leads to the disappearance of HMF signals (spectra 4–7, Fig. 8a) finally resulting in the spectrum of FFCA (spectrum 8 in Fig. 8a). However, when catalase was present, the spectrum of FFCA (5′, Fig. 8b) evolved to the formation of FDCA with a single signal at 6.8 ppm (spectra 6′–8′, Fig. 8b). This signal was slightly displaced with respect to the standard, but addition of external FDCA allowed its unequivocal identification. Therefore, under these conditions, FDCA was the final compound of the reaction.

Quantification of the NMR signals leads to kinetic profiles similar to the ones obtained from the HPLC analyses (Fig. 6a, b), showing the total conversion of HMF into FFCA in the absence of catalase (Additional file 1: Fig. S4A) and the complete conversion of HMF into FDCA when catalase was present in the reaction mixture (Additional file 1: Fig. S4B). The NMR profiles also indicate that the mass balance is maintained throughout the reaction, ruling out the formation of undetected dead-end products.

### Improved FDCA production by AAO mutated variants

**Fig. 9:**
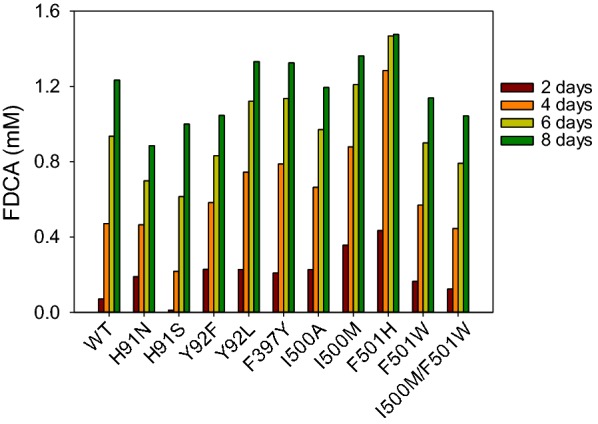
FDCA production by AAO variants after different reaction times. Reactions between 1.5 mM substrate and 1.5 µM AAO in presence of 10 U/mL catalase were performed in 50 mM sodium phosphate, pH 6.0, at 28 °C

The above results first confirm that AAO is able to catalyze the three oxidation steps in the conversion of HMF into FDCA. Second, they reveal that addition of catalase to eliminate the H_2_O_2_ produced during the reactions results in total HMF oxidation to FDCA. AAO has a buried active site in front of the *re* side of the isoalloxazine ring of FAD (Additional file 1: Fig. S5) [23]. Hence, several AAO variants, mutated in residues located at the active site or its access channel (Additional file 1: Fig. S6), were evaluated for the production of FDCA under the above conditions (Fig. 9). The best candidates were Y92L and F397Y with more than 70% FDCA yield and I500M and F501H with 80% and 97% FDCA yields, respectively, after 6 days. These variants showed higher catalytic efficiencies or higher turnover numbers for the transformation of HMF and/or DFF (Additional file 1: Table S2).

The above reaction conditions (1.5 mM of substrate and 1.5 µM of enzyme) are far from being optimal for scaling up the HMF conversion. However, we increased the amount of FDCA obtained by using higher concentrations of HMF (15 mM), trying to attain saturating conditions. Under these conditions, the amount of FDCA produced increased around fivefold for the native enzyme and the F397Y and F501H variants, and up to tenfold for I500M/F501W, reaching total turnover number (TTN) over 16,000 (Table 3).

**Table 3:** Catalytic performance parameters for the production of FDCA from HMF by native AAO, and its F501H, F397Y, and I500M/F501 W variants, in the presence of catalase (10–17 U/mL) in 50 mM sodium phosphate, pH 6.0, at 28 °C, after 6 days of reaction

AAO variant	[HMF] (mM)	% conversion	[FDCA] mM	TTN
WT	1.5	62.4	0.9	1980
	15	26.6	4.2	8440
F501H	1.5	97.9	1.6	3100
	15	43.0	6.8	13,600
F397Y	1.5	75.7	1.2	2400
	15	45.0	7.1	14,300
I500M/F501W	1.5	52.8	0.8	1670
	15	50.8	8.0	16,100

## Discussion

AAO from *P. eryngii* is a model GMC oxidoreductase, whose structural–functional relationships, catalytic mechanisms, and biotechnological interest have been largely investigated [11, 21, 23–25]. In contrast to the results obtained with the related GMC from *Methylovorus* sp. HMFO [16, 17], previous studies had suggested that the AAO from *P. eryngii* was unable to oxidize FFCA. As a consequence, a combination of AAO with *A. aegerita* UPO was used for the complete conversion of HMF into FDCA [13].

Here, we demonstrate that AAO is able to oxidize FFCA despite the fact that the conversion from HMF stops at the FFCA level. The main difference between the conversion of added FFCA and the conversion of the FFCA formed from HMF is the two equivalents of H_2_O_2_ present in the latter case. The results of AAO incubation with H_2_O_2_ showed that the enzyme is relatively stable against peroxide, but its reaction on FFCA is inhibited by all peroxide concentrations (≥ 12.5 µM). Such inhibitory effect is confirmed by catalase addition to the AAO reactions with HMF (and DFF), which results in FDCA production. Therefore, the addition of catalase enables the complete oxidation of HMF to FDCA by *P. eryngii* AAO, as reported for other reactions catalyzed by GMC oxidases [26, 27]. The presence of an unstable dead-end product not detected under the HPLC conditions was ruled out by monitoring the HMF reactions (with/without catalase) directly in the NMR tube (with DFF/FFCA as the sole products along the catalase-free reaction, and DFF/FFCA/FDCA when catalase was added to the reaction).

Concerning the AAO oxidation of FFCA, kinetic studies, in combination with the chromatographic and NMR analyses of the reaction products, suggest that the reaction proceeds by a mechanism not described so far. In the case of HMF and DFF, the enzyme is first reduced by hydride abstraction from the substrate, and then re-oxidized by O_2_ reduction releasing H_2_O_2_. However, during the direct oxidation of FFCA, full conversion to FDCA without H_2_O_2_ formation is observed. Similar monooxygenase activity has been reported in cholesterol oxidase, another member of the GMC superfamily, during hydroxylation of steroids [28]. FFCA conversion into FDCA by a comparable mechanism would be in agreement with the nearly negligible hydration degree of the former compound [13], a requisite for aldehyde oxidation by the AAO hydride abstraction mechanism [12].

Regarding the inhibition of the FFCA oxidation by the presence of H_2_O_2_, it is necessary to mention that a similar effect has been reported for glucose oxidase, another closely related GMC oxidase. Such inhibition is 100-fold faster during the enzyme oxidative half-reaction [29] and results from the strong competition of H_2_O_2_ with O_2_ [30]. In HMF conversion by AAO, the first two oxidation steps are comparatively fast (*k*_1_ and *k*_2_ of 2.5 and 1.4 h^−1^, respectively) and no inhibitory effect of the H_2_O_2_ formed (or exogenously added) is observed. In contrast, the very slow FFCA conversion (*k*_3_ of 0.016 h^−1^) requires × 100 reaction time (with the enzyme remaining under turnover conditions) and would result in the competitive inhibition of AAO by H_2_O_2_ (at concentrations as low as 12.5 µM, under the present conditions).

In addition to demonstrating that the AAO inability to fully oxidize HMF up to FDCA is due to the effect of the H_2_O_2_ formed in the first two oxidation steps, a procedure for the complete oxidation of HMF to FDCA by *P. eryngii* AAO in the presence of catalase, as reported for other reactions catalyzed by GMC oxidases [26, 27], is described here. Moreover, taking advantage of the beneficial effect of catalase on the HMF reactions, we were able to identify several directed variants in residues of the AAO active site. Those resulted in up to sixfold higher conversion to FDCA, in 2-d HMF reactions, than the native enzyme. This increase in FDCA production is justified by a higher relative activity on furfural aldehydes with regard to furfural alcohols, as shown by the *k*_cat_^DFF^/*k*_cat_^HMF^ ratio referred to the native enzyme (Additional file 1: Table S2) that increased up to 1.7-, 3.8-, and 10.7-fold, respectively, for F397Y, I500M/F501 W, and F501H, three of the variants that yielded higher FDCA production (Table 3 and Fig. 9).

## Conclusions

Here, we demonstrate how AAO can act on the aldehyde group of FFCA to produce FDCA. The lack of activity on this compound suggested in previous studies is caused by an inhibitory effect of the H_2_O_2_ formed during the previous oxidation steps when the starting substrate is HMF (or DFF). The use of catalase to remove the H_2_O_2_ produced throughout the reaction allowed achieving complete HMF oxidation, and the identification of several AAO variants with higher activity on HMF and DFF than the wild-type AAO. These variants, in turn, are able to increase the production of FDCA in shorter reaction times.

## Methods

### Chemicals

HMF was kindly provided by AVA Biochem. HMFCA, DFF, FDCA, *p*-methoxybenzyl alcohol, *p*-anisaldehyde (= *p*-methoxybenzaldehyde), catalase from bovine liver (2000–5000 U/mg), and D_2_O were purchased from Sigma-Aldrich (St. Louis, MO, USA). FFCA was purchased from TCI America (Portland, OR, USA). AmplexRed^®^ and horseradish peroxidase (HRP) were obtained from Invitrogen (Walthem, MA, USA). H_2_O_2_ was purchased from Merck (Darmstadt, Hessen, Germany).

### Enzyme production and purification

Native AAO from *P. eryngii* was obtained by expression of the mature AAO cDNA (GenBank AF064069) in *E. coli* followed by in vitro activation and purification as previously described [31].

Variants were produced by site-directed mutagenesis using synthetic primers (Additional file 1: Table S3) and the mutations were confirmed by gene sequencing. Variants were purified to electrophoretic homogeneity following the same protocol as for the native protein [31].

The concentrations of AAO and its variants were estimated in a Cary-100Bio spectrophotometer using their extinction coefficients (Additional file 1: Table S4), either taken from the literature, or calculated in the present work by heat denaturation [32] using *ε*_450_ = 11,300 M^−1^ cm^−1^ for the free FAD [33].

### AAO stability against H_2_O_2_

The AAO stability against H_2_O_2_ was estimated by pre-incubating the enzyme (~ 3 μM) in 50 mM sodium phosphate, pH 6.0, with different H_2_O_2_ concentrations (from 0 to 7.5 mM). Residual activities were estimated by oxidation of saturating concentrations of *p*-methoxybenzyl alcohol in 50 mM sodium phosphate, pH 6.0, at room temperature, immediately after mixing and after 24-h, 48-h, 72-h, and 96-h incubation at 25 °C. The highest activity after mixing was taken as 100% activity, and the percentages of residual activity at the different times and H_2_O_2_ concentrations were calculated according to this maximal value.

### Kinetic analyses

Steady-state parameters for the AAO oxidation of *p*-methoxybenzyl alcohol were calculated by monitoring spectrophotometrically the initial rate of the oxidation of the alcohol to the corresponding aldehyde (Δ*ε*_285_ = 16,950 M^−1^cm^−1^) [34] at 25 °C in air-saturated 50 mM sodium phosphate, pH 6.0. Different amounts of H_2_O_2_ were added to assess its inhibitory effect. To evaluate the possible inhibitory effect of FFCA, the kinetic parameters for the oxidation of *p*-methoxybenzyl alcohol were calculated by monitoring the production of H_2_O_2_ using an HRP-coupled assay with Amplex-Red^®^ (Δ*ε*_563_ = 52,000 M^−1^cm^−1^) as the final substrate. In both cases, apparent kinetic parameters in atmospheric oxygen were determined by fitting the initial reaction rates at different alcohol concentrations to the Michaelis–Menten equation:

1 $$\frac{v}{e} = \frac{{k_{\text{cat}} \left[ A \right]}}{{K_{m} + \left[ A \right]}}$$

On the other hand, oxidation of *p*-anisaldehyde was measured spectrophotometrically by monitoring the initial formation rate of the corresponding acid using the calculated difference in the molar absorbance coefficients of the aldehyde and the acid (*ε*_acid_ − *ε*_aldehyde_, Δ*ε*_247_ = 7139 M^−1^cm^−1^) at 25 °C in air-saturated 50 mM sodium phosphate, pH 6.0, in cuvettes of 1 mm of path length. To evaluate the inhibitory effect of H_2_O_2_, different amounts (0–7.5 mM) were added to the reaction mixture with saturating concentration of aldehyde.

### Long-term oxidation of HMF, DFF, or FFCA

FDCA production by AAO was assayed using 1.5–2.5 µM enzyme with 1.5 mM HMF, DFF, or FFCA in 50 mM sodium phosphate, pH 6.0, at 28 °C, and 300 rpm shaking. Samples were taken at different times and the reaction was stopped by adding 1 M HCl up to pH 2–3. The products of the reactions were analyzed by HPLC, using an ion-exchange Hi-Plex H column (300 × 7.7 mm, 8 µm of particle size, Agilent Technologies). Compounds were eluted with 10 mM H_2_SO_4_ as mobile phase, at a flow rate of 0.8 mL/min, and 60 °C. The retention times of FDCA, HMFCA, FFCA, HMF, and DFF were 14, 17, 19, 24, and 29 min, respectively (Additional file 1: Fig. S7A). Detection of the different compounds was done at 264 nm and their quantification was performed by the corresponding calibration curves (Additional file 1: Fig. S7B). As controls, solutions of the substrates were treated in the same way but in the absence of enzyme to monitor eventual spontaneous oxidation, and in the presence of catalase to check a possible oxidase activity.

AAO residual activity and the amount of H_2_O_2_ released along the reactions were measured by taking aliquots before addition of HCl. Amplex-Red^®^, in combination with HRP, was used to quantify the H_2_O_2_ released. In the presence of HRP, the Amplex-Red^®^ reagent reacts with H_2_O_2_ in a 1:1 stoichiometry to produce resorufin, a colored compound with *ε*_563_ = 52,000 M^−1^cm^−1^. Quantification was performed using a calibration curve with known concentrations of H_2_O_2_. AAO residual activity was tested by measuring its activity against *p*-methoxybenzyl alcohol along the reaction, and these experimental values were fitted to Eq. 2 describing the enzymatic activity loss as a function of time. This allowed the estimation of the AAO half-life for each process (Eq. 3):

2 $${\text{AAO}}_{\text{activity}} \left( \% \right) = {\text{AAO}}_{{{\text{act}}{\kern 1pt} 0}} e^{ - \lambda t}$$

3 $$t_{{\frac{1}{2}}} = \frac{{{ \ln }2}}{\lambda }$$

### NMR assays

Reactions between AAO and HMF, either in the absence or presence of catalase, were monitored by NMR spectroscopy. Reactions were performed in 5 mm NMR tubes in 50 mM sodium phosphate, pH 6.0, with 10% deuterated water (D_2_O) at 298 K. The course of the reactions was monitored by acquiring ^1^H-NMR spectra at different reaction times until the complete conversion was achieved. NMR spectra were recorded by introducing the tube at the different reaction times in a Bruker (Billenica, MA, USA) Avance 500 MHz instrument with the probe (triple resonance TXI) at 288 K to shift the ^1^H-NMR water signal for avoiding overlapping with the signal of the hydroxymethyl group of HMF. The spectra were referenced with water signal at 4.69 ppm. Spectra of the different furfurals were also acquired at 288 K. The amount of each compound was calculated by integrating characteristic signals at: 4.5, 6.4, and 9.2 ppm for HMF; 5.9, 7.4, and 9.5 ppm for DFF, 6.3 and 9.4 ppm for FFCA, and 6.8 ppm for FDCA (Additional file 1: Fig. S3).

## Supplementary information

**Additional file 1: Table S1.** It includes H_2_O_2_ effect on oxidation of furfurals. **Table S2.** Kinetic parameters of AAO variants. **Table S3.** Sequences of PCR primers. **Table S4.** Spectroscopic properties of AAO variants. **Fig. S1.** Effect of low H_2_O_2_ concentrations on FFCA oxidation. **Fig. S2.** Evolution of HMF, DFF and FFCA controls along time. **Fig. S3.** ^1^H-NMR spectra of furfural standards. **Fig. S4.** NMR time-course of HMF reactions. **Fig. S5.** Surface access to AAO active site. **Fig. S6.** Active site of native AAO and ten variants. **Fig. S7.** HPLC analyses of furfural standards.

## Data Availability

The data sets supporting the conclusions of this article are included within the article (and its additional file).

## Abbreviations

AAO: aryl-alcohol oxidase
CPO: chloroperoxidase
FDCA: 2,5-furandicarboxylic acid
FFCA: 5-formylfurancarboxylic acid
GAO: galactose oxidase
HMF: 5-hydroxymethylfurfural
HMFCA: 5-hydroxymethylfurancarboxylic acid
HRP: horseradish peroxidase
*k*_cat_: catalytic constant
*k*_cat_/*K*_m_: catalytic efficiency
*K*_m_: Michaelis constant
PEF: poly(ethylene-furandicarboxylate)
PET: poly(ethylene-terephthalate)
TTN: total turnover number
UPO: unspecific peroxygenase
