# Supplementary material for: Complete oxidation of hydroxymethylfurfural to furandicarboxylic acid by aryl-alcohol oxidase
Source: Biotechnol Biofuels. 2019 Sep 10;12:217. doi: 10.1186/s13068-019-1555-z (PMC6737615; doi:10.1186/s13068-019-1555-z)
Supplement: Supplementary file 1 — Additional file 1: Table S1. It includes H2O2 effect on oxidation of furfurals. Table S2. Kinetic parameters of AAO variants. Table S3. Sequences of PCR primers. Table S4. Spectroscopic properties of AAO variants. Fig. S1. Effect of low H2O2 concentrations on FFCA oxidation. Fig. S2. Evolution of HMF, DFF and FFCA controls along time. Fig. S3. 1H-NMR spectra of furfural standards. Fig. S4. NMR time-course of HMF reactions. Fig. S5. Surface access to AAO active site. Fig. S6. Active site of native AAO and ten variants. Fig. S7. HPLC analyses of furfural standards. [file 13068_2019_1555_MOESM1_ESM.pdf]

SUPPORTING INFORMATION

Complete oxidation of hydroxymethylfurfural to furandicarboxylic acid by aryl-alcohol oxidase

Ana Serrano[1], Eva Calviño[2], Juan Carro[1], Marisa I. Sánchez-Ruiz[1], F. Javier Cañada[2] and Angel T. Martínez[1]

[1] Biotechnology for Lignocellulosic Biomass, Centro de Investigaciones Biológicas, CSIC, Ramiro de Maeztu 9, E-28040 Madrid, Spain
[2] NMR and Molecular Recognition, Centro de Investigaciones Biológicas, CSIC, Ramiro de Maeztu 9, E-28040 Madrid, Spain

Supporting information includes H_2O_2 effect on oxidation of furfurals (**Table S1**), Kinetic parameters of AAO variants (**Table S2**), Sequences of PCR primers **(Table S3**), Spectroscopic properties of AAO variants (**Table S4**), Effect of low H_2O_2 concentrations on FFCA oxidation (**Fig. S1**), Evolution of HMF, DFF and FFCA controls along time (**Fig. S2**), ^{1}H-NMR spectra of furfural standards (**Fig. S3**), NMR time-course of HMF reactions (**Fig. S4**), Surface access to AAO active site (**Fig. S5**), Active site of native AAO and ten variants (**Fig. S6**), and HPLC analyses of furfural standards (**Fig. S7**).

Table S1. H_2O_2 effect in the reaction rates and AAO residual activities oxidizing furfurals

$$\text{HMF} \xrightarrow{k_1} \text{DFF} \xrightarrow{k_2} \text{FFCA} \xrightarrow{k_3} \text{FDCA}$$

**Furfural**	**[H_2O_2] (mM)**	**k_1 (h^{-1})**	**k_2 (h^{-1})**	**k_3 (h^{-1})**	**Activity (%)**[a]
HMF[b]	0	2.54 ± 0.03	1.38 ± 0.17	n.d.[e]	93
	1.5	2.61 ± 0.05	1.13 ± 0.06	n.d.	98
	3.0	2.57 ± 0.02	1.07 ± 0.05	n.d.	96
	6.0	2.65 ± 0.02	1.22 ± 0.06	n.d.	90
DFF[c]	0	--	1.23 ± 0.05	n.d.	99
	1.5	--	1.30 ± 0.03	n.d.	95
	3.0	--	1.30 ± 0.03	n.d.	83
	6.0	--	1.34 ± 0.07	n.d.	99
FFCA[d]	0	--	--	0.016 ± 0.002	15
	1.5	--	--	n.d.	32
	3.0	--	--	n.d.	33
	6.0	--	--	n.d.	30

[a] Residual activity measured after 6 h and 7 d of HMF/DFF and FFCA reactions, respectively

[a] k_1 and k_2 were calculated from equation $[DFF] = [HMF]_0(\frac{k_1}{(k_2-k_1})(e^{-k1t} - e^{-k_2 t})$

[b] k_2 was calculated from equation $[DFF] = [DFF]_0 e^{-k_2 t}$

[c] k_3 was calculated from equation $[FFCA] = [FFCA]_0 e^{-k_3 t}$

[e] n.d.: Reaction not detected

Table S2. Kinetic parameters for the oxidation of HMF and DFF by native AAO and different mutated variants[a]

	HMF			DFF			$k_{cat}^{DFF}/k_{cat}^{HMF}$
	k_{cat} (min^{-1})	K_m (mM)	k_{cat}/K_m ($min^{-1}mM^{-1}$)	k_{cat} (min^{-1})	K_m (mM)	k_{cat}/K_m ($min^{-1}mM^{-1}$)	(relative to AAO)
AAO[b]	20.1 ± 0.6	1.6 ± 0.2	12.9 ± 1.2	31.4 ± 0.7	3.3 ± 0.2	9.4 ± 0.5	1.56 (1)
H91N	419.0 ± 14.0	7.3 ± 0.7	57.2 ± 5.9	256.0 ± 15.0	14.5 ± 1.5	17.6 ± 2.1	0.61 (0.39)
H91S	8.9 ± 0.2	5.1 ± 0.3	1.7 ± 0.1	26.3 ± 1.7	11.6 ± 2.3	2.3 ± 0.5	2.96 (1.89)
Y92F	278.0 ± 16.0	47.8 ± 8.5	5.8 ± 0.7	325.0 ± 9.0	39.3 ± 2.9	8.2 ± 0.4	1.17 (0.75)
Y92L	59.8 ± 2.7	6.6 ± 0.9	9.1 ± 1.4	109.0 ± 4.0	16.3 ± 1.7	6.7 ± 0.7	1.82 (1.17)
F397Y	109.0 ± 3.0	1.4 ± 0.2	76.8 ± 10.2	287.0 ± 8.0	7.0 ± 0.6	41.0 ± 3.7	2.63 (1.69)
I500A	4.4 ± 0.2	2.6 ± 0.5	1.7 ± 0.3	21.2 ± 1.3	3.3 ± 0.7	6.4 ± 1.4	4.82 (3.08)
I500M	4.3 ± 0.1	1.7 ± 0.2	2.6 ± 0.3	23.1 ± 1.2	5.4 ± 0.8	4.3 ± 0.7	5.37 (3.44)
F501H	44.3 ± 0.8	16.2 ± 0.9	2.7 ± 0.2	737.0 ± 55.0	3.5 ± 0.7	214.0 ± 44.0	16.64 (10.65)
F501W	211.0 ± 6.0	12.9 ± 0.9	16.4 ± 1.2	36.7 ± 3.5	16.8 ± 2.8	2.2 ± 0.4	0.17 (0.11)
I500M/ F501W	6.3 ± 0.1	0.7 ± 0.1	8.7 ± 0.7	37.2 ± 0.6	5.0 ± 0.3	7.4 ± 0.4	5.90 (3.78)

[a] Reactions in 50 mM sodium phosphate, pH 6.0, at 25ºC
[b] From Carro *et. al.* [1]

Table S3. Forward primers used for PCR mutagenesis and corresponding templates[a]

	Template	Primer (5'-3')
H91N	native AAO	GGG GGG TCT AGC TCT GTT AAC TAC ATG GTC ATG ATG --
H91S	native AAO	-GG TCT AGC TCT GTT AGC TAC ATG GTC ATG ATG C-- --
Y92F	native AAO	GGG TCT AGC TCT GTT CAC TTC ATG GTC ATG ATG CG- --
Y92L	native AAO	GGG TCT AGC TCT GTT CAC CTC ATG GTC ATG ATG CG- --
F397Y	native AAO	--C TTT TCC AAC CAA TGG TAC CAC CCA GCT ATC CCT CG
I500A	native AAO	GAC AAC GCC AAC ACG GCT TTC CAC CCA GTT GG- --- --
I500M	native AAO	GAC AAC GCC AAC ACG ATG TTC CAC CCA GTT GG- --- --
F501H	native AAO	--C AAC GCC AAC ACG ATT CAC CAC CCA GTT GGA ACG --
F501W	native AAO	GAC AAC GCC AAC ACG ATT TGG CAC CCA GTT GG- --- --
I500M/F501W	F501W	GAC AAC GCC AAC ACG ATG TGG CAC CCA GTT GG- --- --

[a]The substituted nucleotides are in bold and the mutated triplets are underlined

Table S4. Spectroscopic properties of AAO variants in the visible region[a]

	$\lambda^{band\ I}$ (nm)	$\lambda^{band\ II}$ (nm)	$\varepsilon^{band\ I}$ ($M^{-1}cm^{-1}$)	Reference
native AAO	386	463	11050	[2]
H91N	384	463	12825	*here*
H91S	379	458	11629	*here*
Y92F	386	463	10044	[3]
Y92L	386	463	11240	[3]
F397Y	382	462	10415	[4]
I500A	386	457	9925	[5]
I500M	384	458	9609	[5]
F501H	385	461	9229	*here*
F501W	387	462	9944	[6]
I500M/F501W	386	460	9290	[5]

[a] Spectra recorded in 50 mM sodium phosphate, pH 6.0

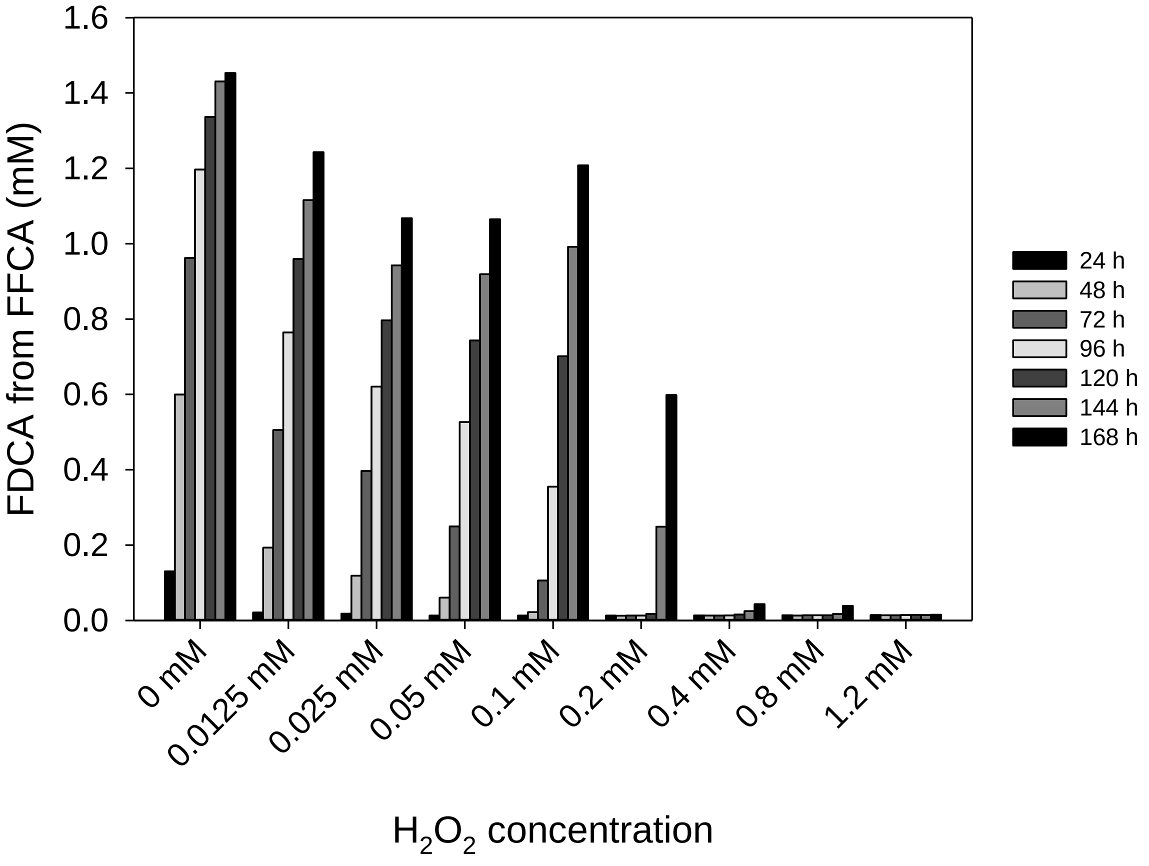

Figure S1. Effect of low H_2O_2 concentrations on the oxidation of FFCA by AAO. Reactions between 1.5 mM FFCA and 2.5 µM AAO were performed in 50 mM sodium phosphate, pH 6.0, at 28ºC in presence of different amounts of H_2O_2 (0.0125 – 1.2 mM).

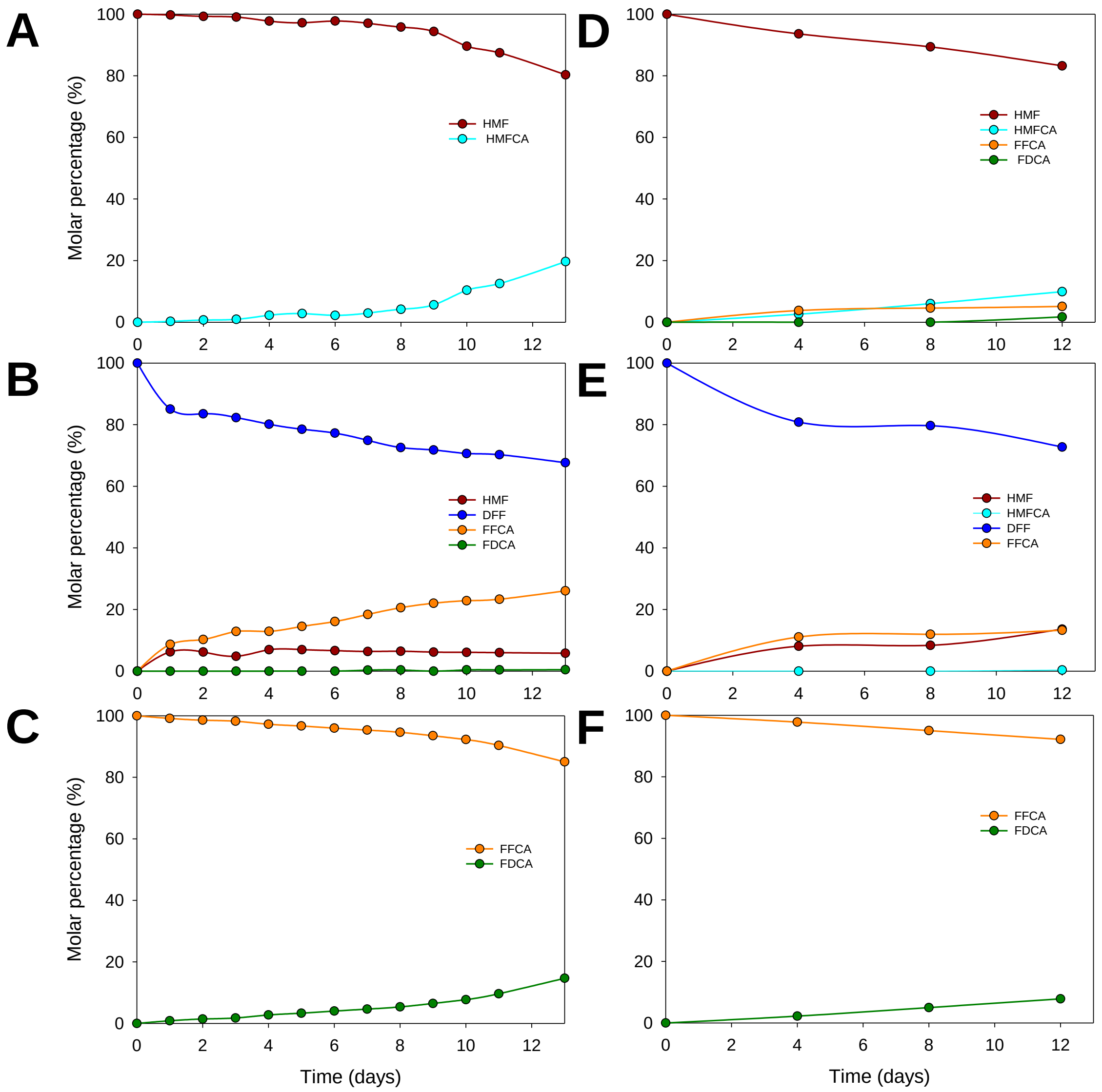

Figure S2. Evolution of HMF (**A** and **D**), DFF (**B** and **E**) and FFCA (**C** and **F**) controls along time in absence of AAO (*left*) and in presence of catalase (10-17 U/mL) (*right*). The three compounds (1.5 mM) were incubated in 50 mM sodium phosphate, pH 6.0, at 28ºC.

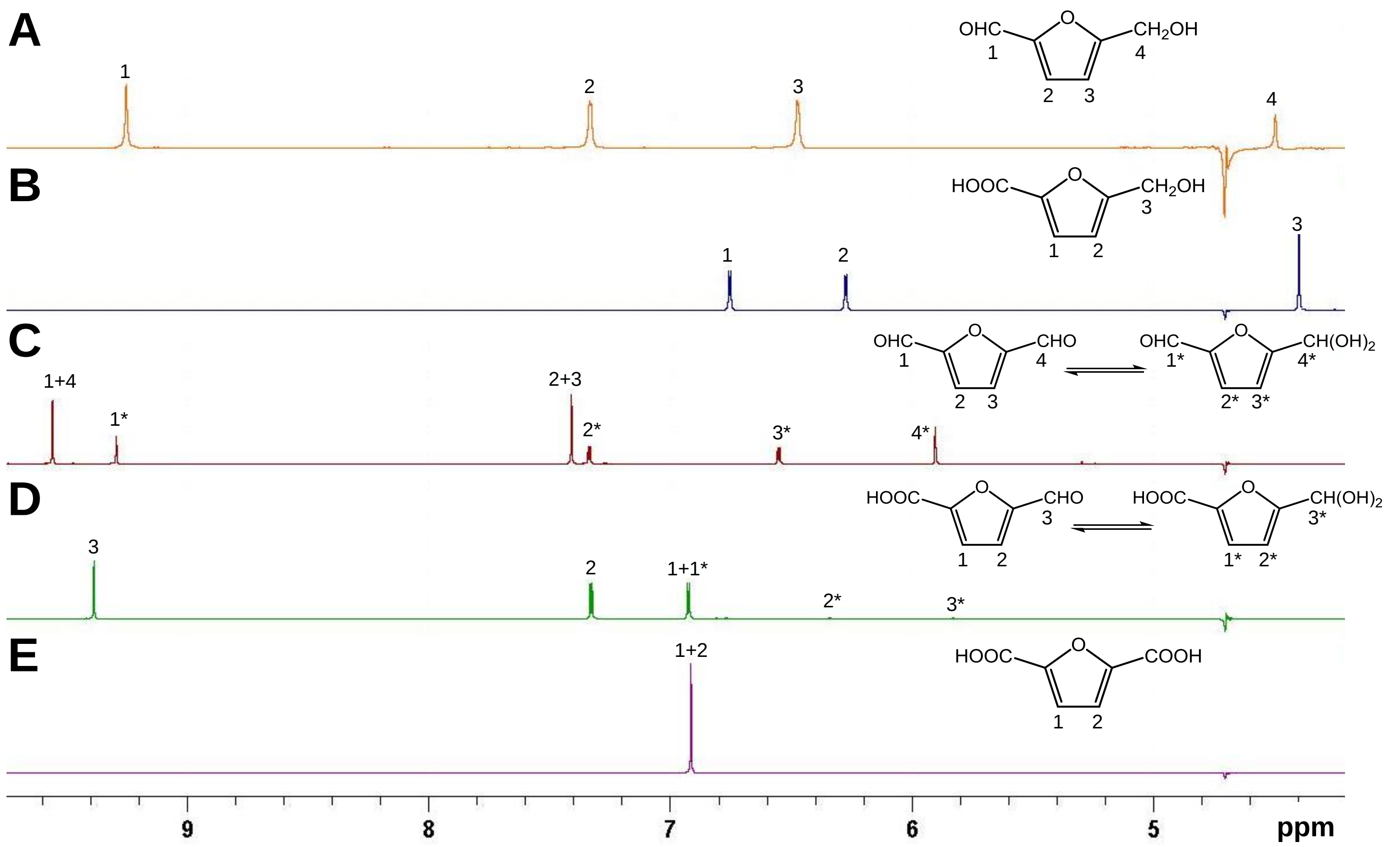

Figure S3. ^{1}H-NMR (4.2-9.8 ppm) analysis of furfural standards: HMF (**A**), HMFCA (**B**), DFF (**C**), FFCA (**D**) and FDCA (**E**) in 50 mM sodium phosphate, pH 6.0, with 10% D_2O. HMF shows four signal for the protons of the aldehyde group (H_1, 9.2 ppm), furanic ring (H_2 and H_3, 7.4 and 6.4 ppm) and hydroxymethyl group (H_4, 4.5 ppm) (**A**). HMFCA shows three signals of the furanic ring (H_1 and H_2, 6.8 and 6.3 ppm) and hydroxymethyl (H_3, 4.4 ppm) protons (**B**). DFF shows signals of: **i)** the aldehyde group (H_1 and H_4, 9.5 ppm) and furanic ring (H_2 and H_3, 7.4 ppm) protons of unhydrated DFF; and **ii)** the unhydrated aldehyde (H_{1*}, 9.3 ppm), furanic ring (H_{2*} and H_{3*}, 7.3 and 6.5 ppm) and hydrated aldehyde (H_{4*}, 5.9 ppm) protons of mono-hydrated DFF. FFCA shows signals of: **i)** the furanic ring (H_1 and H_2, 6.9 and 7.3 ppm) and aldehyde (H_3, 9.4 ppm) protons of unhydrated FFCA; and **ii)** the furanic ring (H_{2*}, 6.3 ppm, and H_{1*}, 6.9 ppm, overlapping with one signal of unhydrated FFCA) and hydrated aldehyde (H_{3*}, 5.8 ppm) protons of hydrated FFCA (with very low intensities due to its low hydration degree) (**D**). FDCA only shows the signal of the furanic ring (H_1 and H_2, 6.9 ppm) protons (**E**).

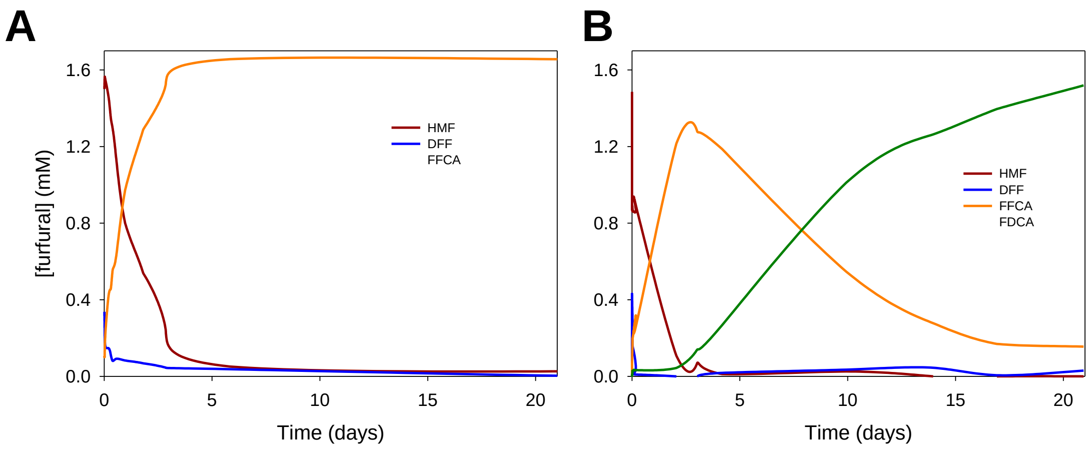

Figure S4. Time course of the reaction of HMF with AAO in absence (**A**) and in presence of catalase (**B**) obtained from NMR experiments. Reactions performed in 50 mM sodium phosphate, pH 6.0, 10% D_2O at room temperature.

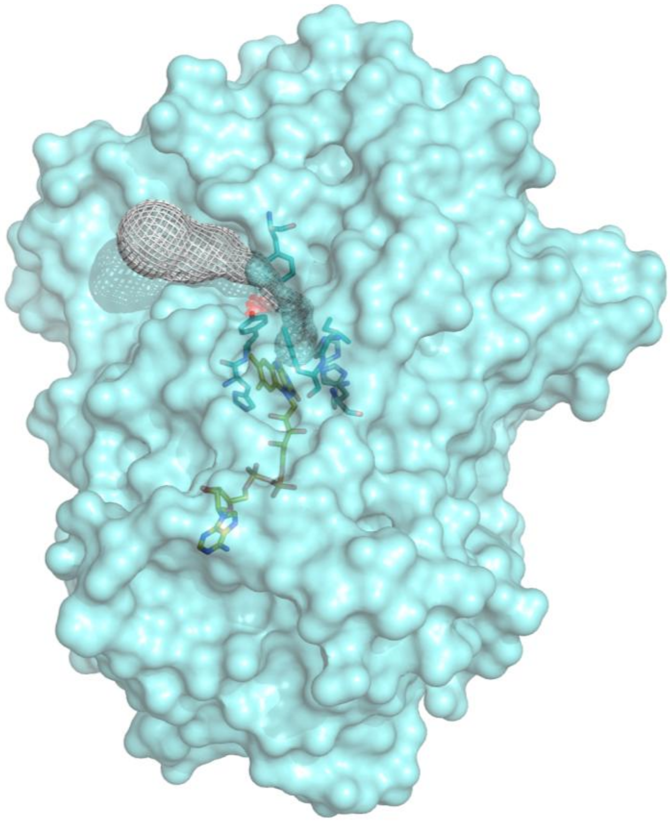

Figure S5. Surface (semi-transparent) access to AAO active site showing the FAD cofactor (green-carbon CPK-colored sticks), neighbor residues (cyan-carbon CPK-colored sticks) and channel to the active site (meshes). Based on PDB ID 3FIM.

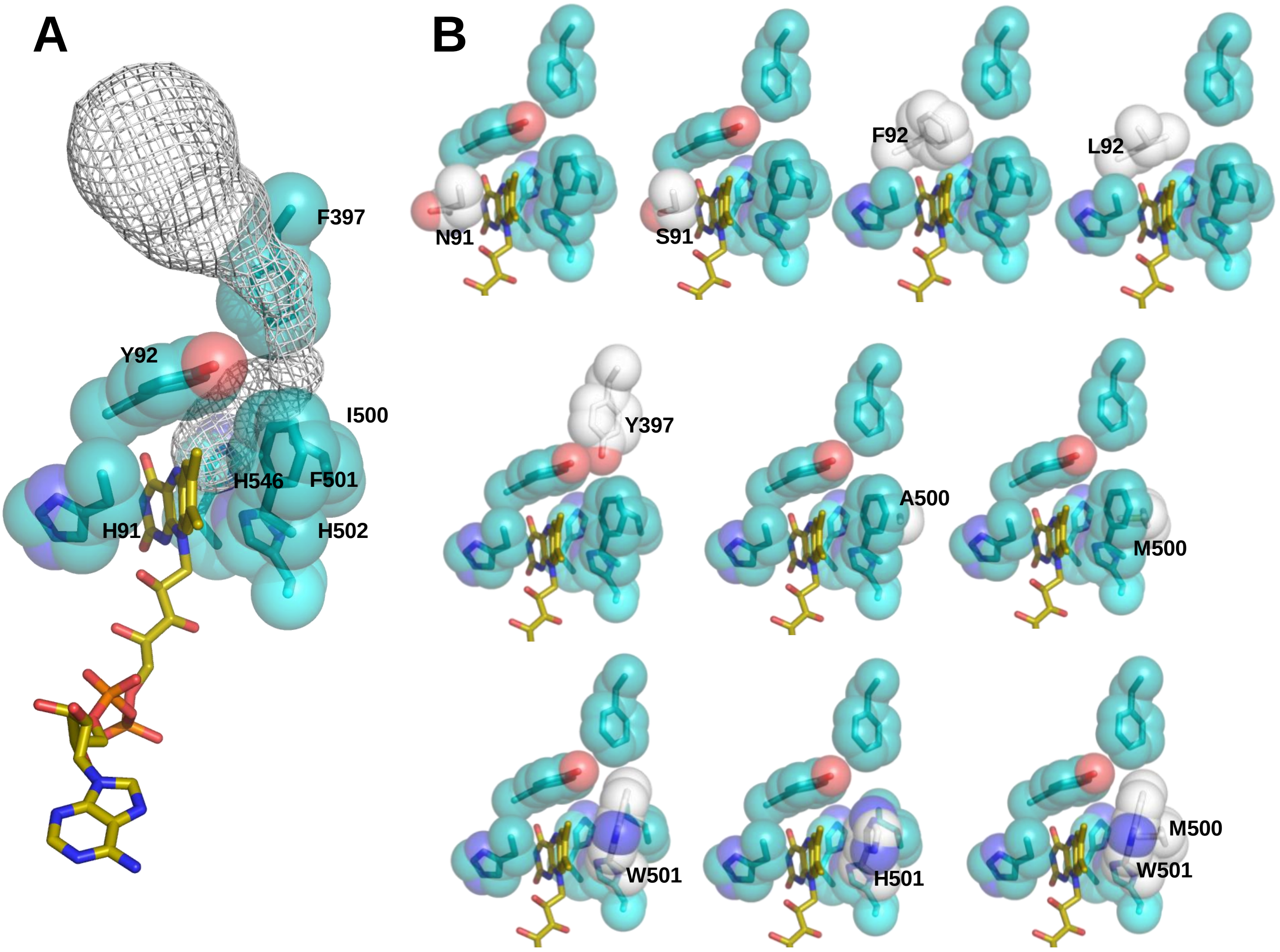

Figure S6. Active site of native AAO (Based on PDB ID 3FIM) (**A**) and ten variants (*in silico* introduced on AAO structure (3FIM) using PyMOL software) assayed for HMF conversion into FDCA (**B**). Residues near the isoalloxazine ring of FAD (gold-carbon CPK-colored sticks) are cyan-carbon CPK-colored (sticks inside semi-transparent spheres) with mutated residues showing white-colored carbons. The access channel (meshes) is also shown in **A**.

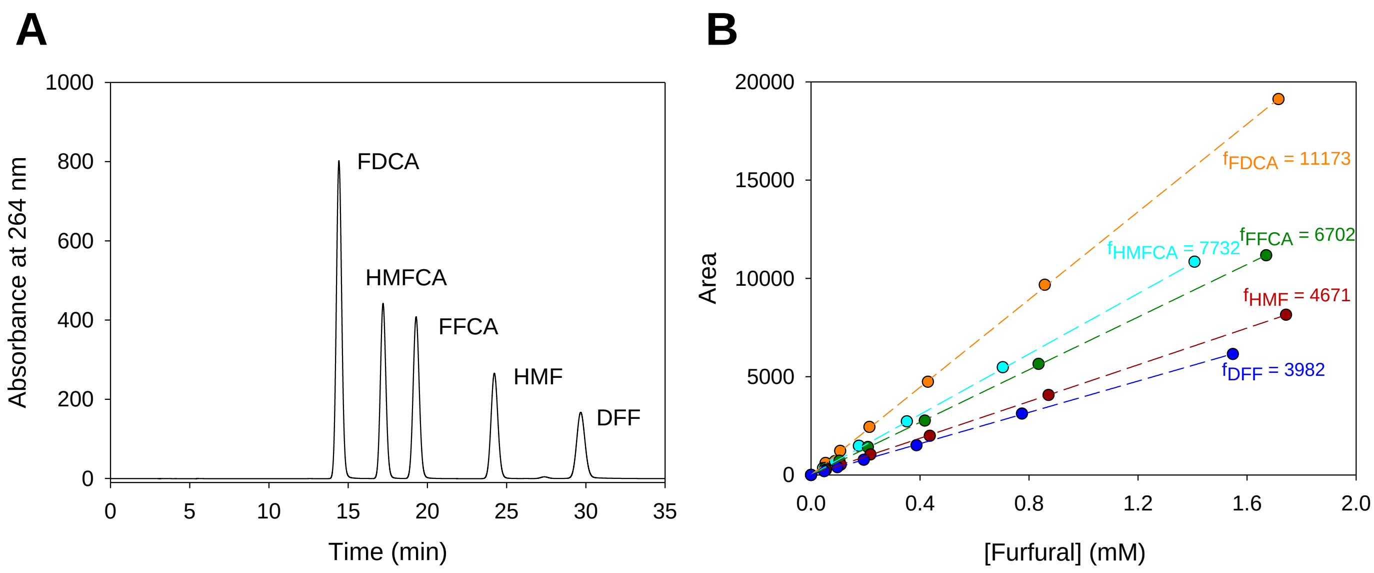

Figure S7. HPLC analysis of furfural standards. (**A**) Elution profile of a mixture of FDCA (t_R, 14 min), HMFCA (t_R, 17 min), FFCA (t_R, 19 min), HMF (t_R, 24 min) and DFF (t_R, 29 min). (**B**) Calibration curve for the different compounds and response factor for the ratio between the absorbance at 264 nm and the concentration.

Supporting references

1. Carro J, Ferreira P, Rodríguez L, Prieto A, Serrano A, Balcells B, Ardá A, Jiménez-Barbero J, Gutiérrez A, Ullrich R, Hofrichter M, Martínez AT. 5-Hydroxymethylfurfural conversion by fungal aryl-alcohol oxidase and unspecific peroxygenase. FEBS J. 2015, 282:3218-29.
2. Ruiz-Dueñas FJ, Ferreira P, Martínez MJ, Martínez AT. In vitro activation, purification, and characterization of *Escherichia coli* expressed aryl-alcohol oxidase, a unique H2O2-producing enzyme. Protein Express Purif. 2006, 45:191-9.
3. Ferreira P, Hernández-Ortega A, Borrelli K, Lucas F, Herguedas B, Guallar V, Martínez AT, Medina M. Aromatic stacking interactions govern catalysis in aryl-alcohol oxidase. FEBS J. 2015, 282:3091-106.
4. Carro J, Amengual-Rigo P, Sancho F, Medina M, Guallar V, Ferreira P, Martínez AT. Multiple implications of an active site phenylalanine in the catalysis of aryl-alcohol oxidase. Sci Rep. 2018, 8:8121.
5. Serrano A, Sancho F, Viña-Gonzalez J, Carro J, Alcalde M, Guallar V, Martínez AT. Switching the substrate preference of fungal aryl-alcohol oxidase: towards stereoselective oxidation of secondary benzyl alcohols. Catal Sci Technol. 2019, 9:833-41.
6. Hernández-Ortega A, Lucas F, Ferreira P, Medina M, Guallar V, Martínez AT. Modulating O2 reactivity in a fungal flavoenzyme: Involvement of aryl-alcohol oxidase Phe-501 contiguous to catalytic histidine. J Biol Chem. 2011, 286:41105-14.
